# Supplementary material for: Chemosensor receptors are lipid-detecting regulators of macrophage function in cancer
Source: Nat Immunol. 2025 Jun 30;26(7):1182–97. doi: 10.1038/s41590-025-02191-x (PMC12208882; doi:10.1038/s41590-025-02191-x)
Supplement: Supplementary file 1 — Reporting Summary [file 41590_2025_2191_MOESM1_ESM.pdf]

nature portfolio

Corresponding author(s): Diletta Di Mitri

Last updated by author(s): 26/02/2025

Reporting Summary

Nature Portfolio wishes to improve the reproducibility of the work that we publish. This form provides structure for consistency and transparency in reporting. For further information on Nature Portfolio policies, see our Editorial Policies and the Editorial Policy Checklist.

Statistics

For all statistical analyses, confirm that the following items are present in the figure legend, table legend, main text, or Methods section.

n/a	Confirmed	
☐	☒	The exact sample size (*n*) for each experimental group/condition, given as a discrete number and unit of measurement
☐	☒	A statement on whether measurements were taken from distinct samples or whether the same sample was measured repeatedly
☐	☒	The statistical test(s) used AND whether they are one- or two-sided<br>*Only common tests should be described solely by name; describe more complex techniques in the Methods section.*
☒	☐	A description of all covariates tested
☐	☒	A description of any assumptions or corrections, such as tests of normality and adjustment for multiple comparisons
☐	☒	A full description of the statistical parameters including central tendency (e.g. means) or other basic estimates (e.g. regression coefficient) AND variation (e.g. standard deviation) or associated estimates of uncertainty (e.g. confidence intervals)
☐	☒	For null hypothesis testing, the test statistic (e.g. *F*, *t*, *r*) with confidence intervals, effect sizes, degrees of freedom and *P* value noted<br>*Give P values as exact values whenever suitable.*
☒	☐	For Bayesian analysis, information on the choice of priors and Markov chain Monte Carlo settings
☒	☐	For hierarchical and complex designs, identification of the appropriate level for tests and full reporting of outcomes
☐	☒	Estimates of effect sizes (e.g. Cohen's *d*, Pearson's *r*), indicating how they were calculated

Our web collection on statistics for biologists contains articles on many of the points above.

Software and code

Policy information about availability of computer code

Data collection

Gepia2 software was utilized to analyze gene expression data from the TGCA dataset.

Data analysis

RNA-seq and bioinformatics analysis were performed to analyze the gene expression profiles of the samples. The following steps were carried out:

Sequencing and Quality Control:
All samples were sequenced on an Illumina NextSeq500 platform, generating an average of 15 million 75-bp single-end reads per sample. After sequencing, quality control checks were performed to assess the data quality and remove low-quality reads or artifacts.

Alignment and Read Counting:
The quality-filtered reads were aligned to the human genome (GRCm38) using the STAR aligner with default parameters (version 2.6.1). The aligned reads were then used to obtain gene-based read counts using the featureCounts module (version 1.6.4) and the Ensembl GRCm38 annotation.

Normalization and Differential Gene Expression Analysis:
To compare expression levels across samples, raw read counts were normalized using the TMM (trimmed mean of log-ratio values) method. Genes with counts per million (CPM) mapped reads greater than 1 in at least 2 libraries were considered for further analysis. The edgeR package (version 3.26.5) in R statistical software was used to perform differential gene expression analysis.

CRISPR-Cas9 Analysis:
To analyze the DNA sequencing data derived from the CRISPR-Cas9 library, the software MAGECK (Model-based Analysis of Genome-wide

CRISPR-Cas9 Knockout) was utilized.

For manuscripts utilizing custom algorithms or software that are central to the research but not yet described in published literature, software must be made available to editors and reviewers. We strongly encourage code deposition in a community repository (e.g. GitHub). See the Nature Portfolio guidelines for submitting code & software for further information.

Data

Policy information about availability of data

All manuscripts must include a data availability statement. This statement should provide the following information, where applicable:

- Accession codes, unique identifiers, or web links for publicly available datasets
- A description of any restrictions on data availability
- For clinical datasets or third party data, please ensure that the statement adheres to our policy

A data avaiability statment has been included

Research involving human participants, their data, or biological material

Policy information about studies with human participants or human data. See also policy information about sex, gender (identity/presentation), and sexual orientation and race, ethnicity and racism.

Reporting on sex and gender	Enrolled patients were all male considering the nature of the study (prostate cancer)
Reporting on race, ethnicity, or other socially relevant groupings	No socially relevant groupping was applied to the study
Population characteristics	Prostate cancer patients that received biopsy for their clinical practice, in absence of prior therapy.
Recruitment	Tissue samples were retrieved from routine biopsies of patients diagnoses with PCa
Ethics oversight	The protocol was approved by the Ethical Commitee of Humanitas Clinical and research Hospital

Note that full information on the approval of the study protocol must also be provided in the manuscript.

Field-specific reporting

Please select the one below that is the best fit for your research. If you are not sure, read the appropriate sections before making your selection.

☒ Life sciences ☐ Behavioural & social sciences ☐ Ecological, evolutionary & environmental sciences

For a reference copy of the document with all sections, see nature.com/documents/nr-reporting-summary-flat.pdf

Life sciences study design

All studies must disclose on these points even when the disclosure is negative.

Sample size	In most experiments sample size was calculated with a simple size calculator, basing on preliminary data
Data exclusions	No data were excluded
Replication	Experiments were repeated and the number of each experiment is reported in the figure legend
Randomization	Samples, mice and human samples were randomly assigned to experimental groups
Blinding	Measurement of the tumour size were performed in blind

Reporting for specific materials, systems and methods

We require information from authors about some types of materials, experimental systems and methods used in many studies. Here, indicate whether each material, system or method listed is relevant to your study. If you are not sure if a list item applies to your research, read the appropriate section before selecting a response.

Materials & experimental systems

n/a	Involved in the study	
☐	☒	Antibodies
☐	☒	Eukaryotic cell lines
☒	☐	Palaeontology and archaeology
☐	☒	Animals and other organisms
☒	☐	Clinical data
☒	☐	Dual use research of concern
☒	☐	Plants

Methods

n/a	Involved in the study	
☒	☐	ChIP-seq
☐	☒	Flow cytometry
☒	☐	MRI-based neuroimaging

Antibodies

Antibodies used

Antibody Fluorochrome Source Identifier Application Dilution
B-actin unconjugated Abcam ab213262 Western Blot 1:1000
STAT6 unconjugated Cell Signaling 9362 Western Blot 1:1000
anti-rabbit secondary antibody HRP-conjugated Bio-Techne HAF008 Western Blot 1:1000
anti-mouse secondary antibody HRP-conjugated Bio-Techne HAF007 Western Blot 1:1000
CD45 BUV563 BD 612924 FACS 1 uL in 100
KLRG1 PerCPCy5.5 BD 563595 FACS 0.63 uL in 100
CD39 PECy7 Biolegend 143806 FACS 0.31 uL in 100
CD4 BUV496 BD 612952 FACS 1.25 uL in 100
CD8 BUV805 BD 612898 FACS 0.63 uL in 100
NK1.1 BUV395 BD 564144 FACS 0.63 uL in 100
CD45R BV570 Biolegend 103237 FACS 1.25 uL in 100
CD127 PECy5 Biolegend 135016 FACS 2.5 uL in 100
CD62L BV510 Biolegend 104441 FACS 1.25 uL in 100
CD44 PEDazzle594 Biolegend 103056 FACS 0.63 uL in 100
CD25 AF488 Biolegend 102017 FACS 0.63 uL in 100
PD1 (CD279) BV605 Biolegend 135220 FACS 2.5 uL in 100
TIM3 BV711 Biolegend 119727 FACS 1.25 uL in 100
Ki67 AF647 Biolegend 151206 FACS 0.5 uL in 100
FOXP3 BV421 Biolegend 126419 FACS 1.25 uL in 100
Ly6G BV785 Biolegend 127645 FACS 0.15 uL in 100
Ly6C APC Cy7 Biolegend 128026 FACS 0.15 uL in 100
MHC II BV480 BD 566086 FACS 0.15 uL in 100
CD206 AF647 Biolegend 141712 FACS 1.25 uL in 100
CD11b PECF594 BD 562287 FACS 0.15 uL in 100
F480 BV421 Biolegend 123132 FACS 0.63 uL in 100
CD11c PerCPCy5.5 Biolegend 117328 FACS 1.25 uL in 100
CD115 BV711 Biolegend 135515 FACS 1.25 uL in 100
CD3 BV650 Biolegend 100229 FACS 1.25 uL in 100
Arginase-1 AF700 LifeTechnologies 56-3697-80 FACS 1.25 uL in 100
IFNgamma APC Biolegend 505810 FACS 0.63 uL in 100
CD45.1 BV650 BD 563754 FACS 1 uL in 100
CD45.2 PeCy7 Biolegend 109830 FACS 1 uL in 100
CD8 BV605 Biolegend 100744 FACS 1 uL in 100
CD103 PE Biolegend 156904 FACS 2.5 uL in 100

Antibody Metal Source Identifier Application Dilution
KI67 142Nd ABCAM ab279657 IMC 1:100
CD20 176YB BD 555677 IMC 1:100
CD31 153 Eu ABCAM ab226157 IMC 1:100
CD14 145Nd ABCAM ab209971 IMC 1:100
NKp46 156Gd Biotechne AF1850 IMC 1:100
Foxp3 166Er Biolegend 320202 IMC 1:100
HLA-DR 174Yb eBioscience 14-9956-82 IMC 1:200
CD45 152Sm Cell Signalling 47937SF IMC 1:200
CD8 167Er Cell Signalling #90257 IMC 1:100
PD1 165Ho Abcam ab186928 IMC 1:200
MARCO 158Gd LsBio LS-B15577 IMC 1:50
Pan-Cytokeratin 148Nd Abcam ab264485 IMC 1:100
CD68 159Tb Abcam ab233172 IMC 1:100
COLLAGEN TYPE I 169Tm Standard Biotools 3169023D IMC 1:400
CD16 146Nd Abcam ab256582 IMC 1:100
PDL1 150Nd Cell Signalling #85164 IMC 1:50
CD11c 154Sm Abcam ab264107 IMC 1:100
CD3 170Er Standard Biotools 3170019D IMC 1:50
CD66b 161Dy Biolegend 392902 IMC 1:100
CD39 155 Gd Biolegend 328202 IMC 1:100
aSMA 141Pr Abcam ab240654 IMC 1:100

	CD206 168 Er Abcam ab254471 IMC 1:100<br>CD163 147Sm Standard Biotools 3147021D IMC 1:100<br>CD74 144nD Cell Signalling #95154 IMC 1:100<br>CD11b 149sM Abcam ab209970 IMC 1:25
Validation	All antibodies used in the study are commercially available.<br><br>All antibodies used in the study were titrated before use.<br><br>All antibodies have been validated by the commercial manufacturers. Validation data are available on each manufacturer's website.

Eukaryotic cell lines

Policy information about cell lines and Sex and Gender in Research

Cell line source(s)	The Pten-/- Trp53-/- cell line was provided by R.A. DePinho.<br><br>PC3, HEK293T, L929, 4T1, ID8, and THP1 cells are commercially available and were purchased from ATCC.
Authentication	None of the cell lines used in this work were authenticated.
Mycoplasma contamination	All the cell lines used were tested and were Mycoplasma-free.
Commonly misidentified lines<br>(See ICLAC register)	No commonly misidentified lines were used. (See ICLAC register for reference).

Animals and other research organisms

Policy information about studies involving animals; ARRIVE guidelines recommended for reporting animal research, and Sex and Gender in Research

Laboratory animals	Male mice were used in the study.
Wild animals	This study did not involve wild animals.
Reporting on sex	Only male mice were used considering the nature of the study (prostate cancer)
Field-collected samples	No field-collected samples were used in the study
Ethics oversight	Procedures involving animal handiling and care conformed to protocols approved by the Humanitas Clinical and Research Centre and Italian Minister of Health, in compliance with national and international law and policies

Note that full information on the approval of the study protocol must also be provided in the manuscript.

Plants

Seed stocks	*Report on the source of all seed stocks or other plant material used. If applicable, state the seed stock centre and catalogue number. If plant specimens were collected from the field, describe the collection location, date and sampling procedures.*
Novel plant genotypes	*Describe the methods by which all novel plant genotypes were produced. This includes those generated by transgenic approaches, gene editing, chemical/radiation-based mutagenesis and hybridization. For transgenic lines, describe the transformation method, the number of independent lines analyzed and the generation upon which experiments were performed. For gene-edited lines, describe the editor used, the endogenous sequence targeted for editing, the targeting guide RNA sequence (if applicable) and how the editor was applied.*
Authentication	*Describe any authentication procedures for each seed stock used or novel genotype generated. Describe any experiments used to assess the effect of a mutation and, where applicable, how potential secondary effects (e.g. second site T-DNA insertions, mosiacism, off-target gene editing) were examined.*

Flow Cytometry

Plots

Confirm that:

☐ The axis labels state the marker and fluorochrome used (e.g. CD4-FITC).

☒ The axis scales are clearly visible. Include numbers along axes only for bottom left plot of group (a 'group' is an analysis of identical markers).

☒ All plots are contour plots with outliers or pseudocolor plots.

☒ A numerical value for number of cells or percentage (with statistics) is provided.

Methodology

Sample preparation	Primary macrophages were detached from the plate with Accutase Solution (Thermo Fisher Scientific), and nonspecific antibody binding was prevented by incubating cells with an Fc block (TruStain FcX anti-CD16/32, clone 93). Cells were then stained with LIVE/DEAD Fixable Viability Dye eFluor 780 (BioLegend) for 20 min at 4°C, followed by staining with the antibody mix for 30 min at room temperature.<br><br>For ARG1 detection, after extracellular staining, samples were fixed and permeabilized (Intracellular Fixation & Permeabilization Buffer Set; eBioscience) and stained for 30 min at 4°C. Cells were then fixed in 1% PFA.For analysis of tumour-infiltrating leukocytes, tumours were collected, cut into small pieces, and digested with Collagenase I (1 mg/mL for mouse tissue and 0.5 mg/mL for human tissue) for 45 min at 37°C on a rocking platform. After quick digestion in 2.5% Trypsin and DNase I, single-cell suspension was obtained by mechanical dissociation through a syringe needle (18G) and subsequent filtration on a 40-µm cell strainer.<br><br>Cells were stained with LIVE/DEAD Fixable Viability Dye (BioLegend) for 20 min at 4°C, followed by staining with the antibody mix for 30 min at room temperature. After extracellular staining, samples were fixed and permeabilized (Intracellular Fixation & Permeabilization Buffer Set; eBioscience) and stained with intracellular mix for 30 min at 4°C. Cells were then fixed in 1% PFA.
Instrument	Samples were acquired using a BD FACSymphony™ A5 Cell Analyzer.
Software	Data were analyzed using FlowJo software.
Cell population abundance	Cells frequency is shown in the relevant gates. After sorting, a small aliquot of sorted cells was used to determine purity (>90%).
Gating strategy	The gating strategie are shown in relative extended data.

☒ Tick this box to confirm that a figure exemplifying the gating strategy is provided in the Supplementary Information.
