# Supplementary figures and images for: Chemosensor receptors are lipid-detecting regulators of macrophage function in cancer
Source: Nat Immunol. 2025 Jun 30;26(7):1182–97. doi: 10.1038/s41590-025-02191-x (PMC12208882; doi:10.1038/s41590-025-02191-x)

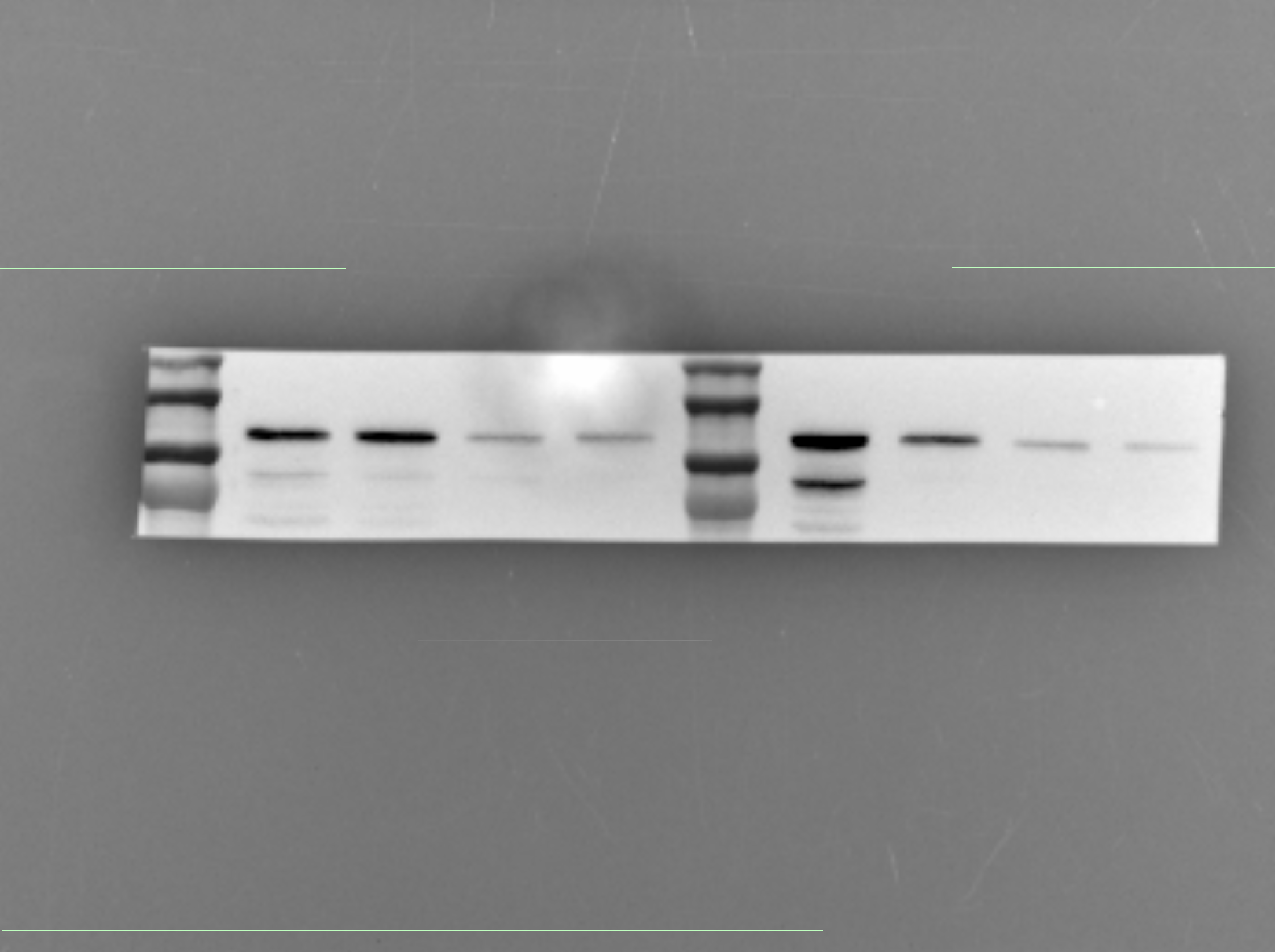

Supplement: Supplementary file 5 — Unprocessed western blot. [file 41590_2025_2191_MOESM5_ESM.tif]

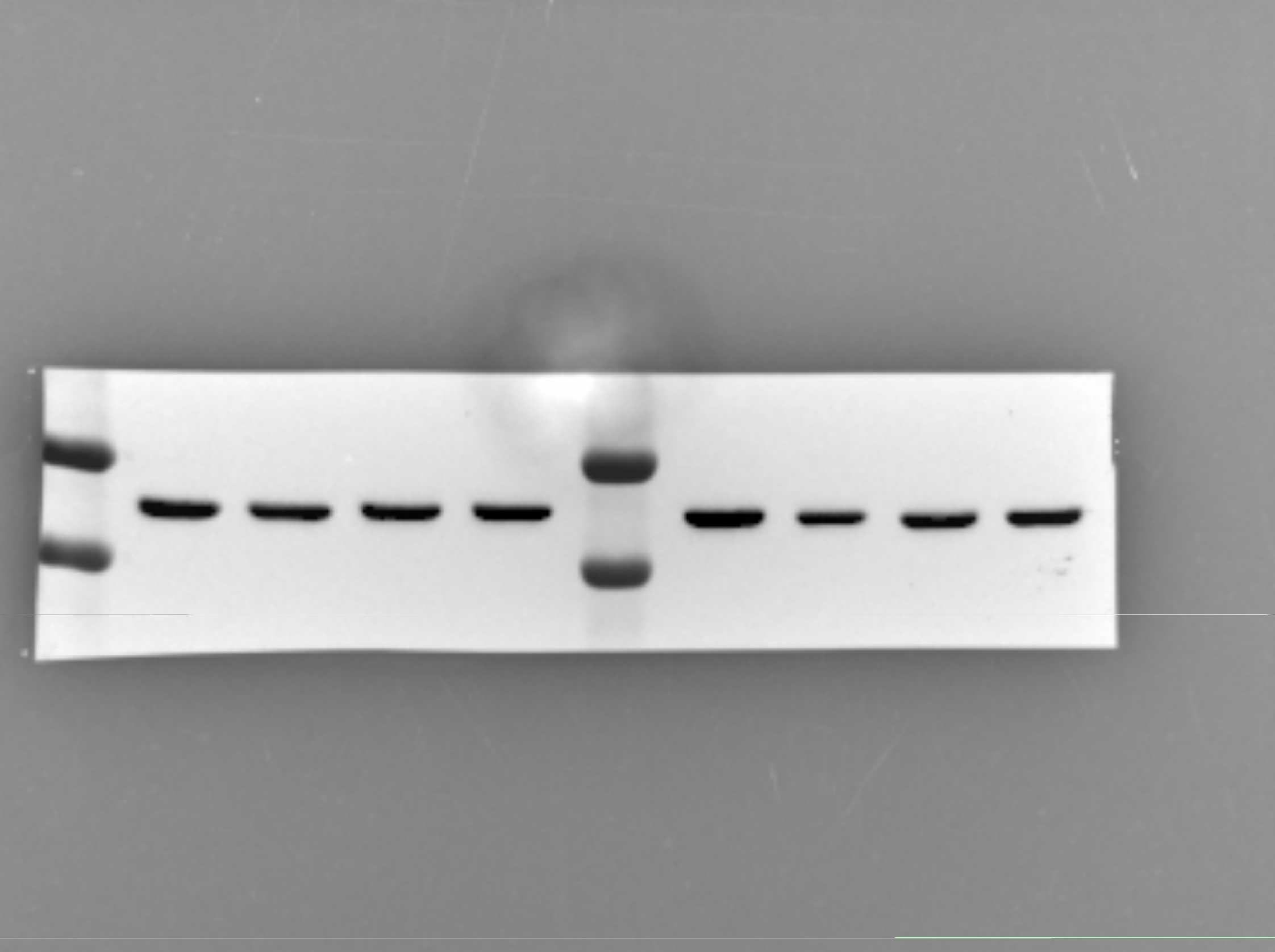

Supplement: Supplementary file 6 — Unprocessed western blot. [file 41590_2025_2191_MOESM6_ESM.tif]
